# New structural forms of a mycobacterial adenylyl cyclase Rv1625c

**DOI:** 10.1107/S2052252514016741

**Published:** 2014-08-22

**Authors:** Deivanayaga Barathy, Rohini Mattoo, Sandhya Visweswariah, Kaza Suguna

**Affiliations:** a Molecular Biophysics Unit, Indian Institute of Science, C. V. Raman Avenue, Bangalore, Karnataka 560 012, India; b Molecular Reproduction, Development and Genetics, Indian Institute of Science, C. V. Raman Avenue, Bangalore, Karnataka 560 012, India

**Keywords:** Rv1625c, adenylyl cyclases, *Mycobacterium tuberculosis*

## Abstract

Rv1625c is one of 16 adenylyl cyclases encoded in the genome of *Mycobacterium tuberculosis*. In solution Rv1625c exists predominantly as a monomer, with a small amount of dimer. It has been shown previously that the monomer is active and the dimeric fraction is inactive. Both fractions of wild-type Rv1625c crystallized as head-to-head inactive domain-swapped dimers as opposed to the head-to-tail dimer seen in other functional adenylyl cyclases. About half of the molecule is involved in extensive domain swapping. The strain created by a serine residue located on a hinge loop and the crystallization condition might have led to this unusual domain swapping. The inactivity of the dimeric form of Rv1625c could be explained by the absence of the required catalytic site in the swapped dimer. A single mutant of the enzyme was also generated by changing a phenylalanine predicted to occur at the functional dimer interface to an arginine. This single mutant exists as a dimer in solution but crystallized as a monomer. Analysis of the structure showed that a salt bridge formed between a glutamate residue in the N-terminal segment and the mutated arginine residue hinders dimer formation by pulling the N-terminal region towards the dimer interface. Both structures reported here show a change in the dimerization-arm region which is involved in formation of the functional dimer. It is concluded that the dimerization arm along with other structural elements such as the N-terminal region and certain loops are vital for determining the oligomeric nature of the enzyme, which in turn dictates its activity.

## Abstract 2

Two new structural forms, a monomer and a swapped dimer, of the catalytic domain of an adenylyl cyclase from *M. tuberculosis* are reported.

## 1. Introduction   

In cell signalling, the importance of second messengers such as cyclic AMP and cyclic GMP in regulating cellular function and homeostasis is well recognized. Adenylyl cyclases convert ATP to cyclic AMP, and these enzymes are classified into six types based on their sequence similarity. Among these six classes of adenylyl cyclases (ACs), class III is the universal class and is present in eukaryotes, bacteria and archaebacteria (Shenoy *et al.*, 2004 ▶). There are about ten isoforms of mammalian adenylyl cyclases, of which nine are membrane-bound (mAC; Sunahara *et al.*, 1996 ▶) and one is cytoplasmic and is therefore referred to as soluble adenylyl cyclase (sAC; Buck *et al.*, 1999 ▶).

Mammalian membrane-bound adenylyl cyclases have a complex structure with 12 transmembrane helices and two cytoplasmic domains. These proteins are arranged in two tandem repeats, with each repeat having six transmembrane helices followed by a cytoplasmic domain. The first cytoplasmic domain is called C1 and the second one is called C2 (Taussig & Gilman, 1995 ▶). The structure of a heterodimer comprised of the C1 and C2 domains crystallized in the presence of the GS α subunit and a forskolin molecule was the first structure of the active cyclase enzyme to be reported (Tesmer *et al.*, 1997 ▶). The C1 and C2 domains in this heterodimer are arranged in a head-to-tail fashion, like a wreath. The C1 domain contributes the metal-binding aspartates and the C2 domain contributes the substrate-specifying and transition-state-stabilizing residues to the active site formed at the dimer interface. Thus, the C1–C2 heterodimer has only one potential active site, with the other site being a pseudo-active site which is occupied by a forskolin molecule. In an earlier report, an inactive homodimer of C2 domains which lacks the residues required for metal binding was shown to form the same type of wreath-like dimeric structure with two bound forskolin molecules (Zhang *et al.*, 1997 ▶). The recent structures of human sAC in complex with a substrate analogue, products and a regulatory bicarbonate molecule revealed the mechanism of catalysis and the activation of sACs by bicarbonate (Kleinboelting *et al.*, 2014 ▶).

Bacteria harbour a number of class III nucleotide cyclases. In contrast to the mACs, the bacterial enzymes have a single catalytic domain that contains all of the residues necessary for substrate binding and catalysis. Thus, these proteins form homodimers with two binding sites at the interface as seen in the structures of *Spirulina platensis* CyaC (sAC; Steegborn *et al.*, 2005 ▶), in two mycobacterial ACs, Rv1264 (Tews *et al.*, 2005 ▶) and Rv1900c (Sinha *et al.*, 2005 ▶), and in the *Pseudomonas aeruginosa* enzyme (Topal *et al.*, 2012 ▶). Crystal structures of the catalytic domains of three soluble guanylyl cyclases (GCs), CYG12 from *Chlamydomonas reinhardtii* (Winger *et al.*, 2008 ▶), Cya2 from *Synechocystis* PCC6803 (Rauch *et al.*, 2008 ▶) and from human (Allerston *et al.*, 2013 ▶), have been reported. One exception to the dimeric form of the cyclase enzyme is the trypanosomal enzyme, which crystallized as a monomer (Bieger & Essen, 2001 ▶) despite being a catalytically active dimer in solution.

Most of the class III ACs have been found to be fused with various other domains which are involved in signal perception and regulation (Shenoy & Visweswariah, 2006 ▶; Linder & Schultz, 2003 ▶). An analysis of the mycobacterial genome by Shenoy *et al.* (2004 ▶) showed the presence of at least 16 class III adenylyl cylases. Crystal structures of the functional dimers of two of these cyclases, Rv1264 and Rv1900c, are available. The structure of Rv1264 revealed that the N-terminal domain regulates the activity of the enzyme in different pH conditions (Tews *et al.*, 2005 ▶) by causing drastic structural changes. In Rv1900c, a loop at the interface with two different conformations in the two protomers of the dimer acts as a regulator by allowing the substrate to enter only one of the two active sites (Sinha *et al.*, 2005 ▶).

The catalytic domain of a third AC from *Mycobacterium tuberculosis*, Rv1625c, has been cloned and demonstrated to be biochemically active (Guo *et al.*, 2001 ▶; Shenoy *et al.*, 2003 ▶). Since it has a slightly higher sequence identity (37%) to mammalian guanylyl cyclases (UniProt ID P51841) than to mammalian adenylyl cyclases (UniProt ID P30803; 35.5%), attempts were made to switch the substrate specificity of this enzyme from ATP to GTP (Shenoy *et al.*, 2003 ▶). In this process several mutants were generated and biochemically characterized, and the crystal structure of one such mutant, the triple mutant KFD→ERC, has been reported (Ketkar *et al.*, 2006 ▶). This mutant crystallized as a monomer and showed significant movements in the loops harbouring the mutated residues, preventing dimer formation and thus making the enzyme catalytically inactive. A homology model of the dimeric structure of Rv1625c based on mACs (PDB entries 1ab8 and 1azs; Zhang *et al.*, 1997 ▶; Tesmer *et al.*, 1997 ▶) revealed the presence of a phenylalanine residue at the subunit interface (Shenoy *et al.*, 2003 ▶). This phenylalanine residue was predicted to be involved in dimer stabilization by forming a stacking interaction (Shenoy *et al.*, 2003 ▶). To further elucidate the role of this residue in dimer stabilization, a single mutant F363R of Rv1625c (Rv1625c-F363R) was generated. This mutant was shown to be dimeric in solution, with a 3000-fold reduced activity (Ketkar *et al.*, 2006 ▶). This may be caused by structural changes at the dimeric interface and/or at the active site. We attempted to crystallize Rv1625c and its mutant Rv1625c-F363R, expecting to obtain crystals of the active dimeric forms of the enzyme. However, Rv1625c-F363R crystallized as a monomer, while the wild-type Rv1625c enzyme crystallized as a dimer with extensive domain swapping. Here, we report these two structures of Rv1625c, which appear to be the forms that the enzyme adopts when it exists in the inactive state. This study also reveals the regulatory roles played by the N-terminal segment and other loops of the structure in modulating the oligomeric state and thus the activity of the enzyme.

## 2. Materials and methods   

### 2.1. Protein preparation   

The catalytic domain of wild-type Rv1625c (Rv1625c-Wt) encompassing residues Met212–Val443 was cloned into pPRO-EX-HT vector to generate a hexahistidine at the N-terminus. The F363R mutant of Rv1625c (Rv1625c-F363R) was generated by performing site-directed mutagenesis as described previously (Shenoy *et al.*, 2003 ▶). Expression and purification of the protein was carried out as described previously (Ketkar *et al.*, 2004 ▶). The protein obtained after Ni–NTA purification was further purified and its oligomeric state was assessed using gel filtration. Gel filtration was performed using a Superose 12 10/300 GL column (Amersham Pharmacia Biotech) equilibrated with buffer consisting of 20 m*M* Tris pH 7.4, 10%(*v*/*v*) glycerol, 5 m*M* β-mercaptoethanol (β-ME), 10 m*M* NaCl on an ÄKTA fast protein liquid chromatography (FPLC) system at a flow rate of 0.25 ml min^−1^. 10% glycerol was required to keep the protein in a stable form. The column was calibrated with standard protein molecular-weight markers from Bio-Rad: bovine γ-globulin (158 kDa), chicken ovalbumin (44 kDa), equine myoglobin (17 kDa) and vitamin B_12_ (1.3 kDa).

### 2.2. Rv1625c-F363R   

#### 2.2.1. Crystallization   

The major peak (dimer) of the single mutant eluted from the gel-filtration column (Supplementary Fig. S1*a*) was concentrated to 3.3 mg ml^−1^ and used for crystallization. Initial screening for crystals was carried out with commercially available crystal screens from Hampton Research by mixing 2 µl protein solution with 2 µl crystallization condition using the microbatch method. Plate-like crystals were obtained in some of the conditions. These conditions were chosen for further optimization. The ammonium acetate that was present in one of the conditions (condition No. 65) in which crystals appeared was replaced by ammonium sulfate and potassium acetate in further setups. After one week, crystals started growing in a condition consisting of 0.15 *M* ammonium sulfate, 0.1 *M* bis-tris pH 5.5, 30% PEG 10K and were allowed to grow further over a period of three weeks before harvesting them.

#### 2.2.2. Data collection   

The crystals (Supplementary Fig. S1*b*) were soaked in a cryoprotectant solution (20% ethylene glycol in the mother liquor) prior to mounting. One of the crystals diffracted to a resolution of 2.05 Å at the home-source X-ray facility with Cu *K*α radiation generated using a rotating-anode X-ray generator (Bruker Microstar) and focused with Osmic mirrors. 180 frames were collected with a crystal-to-detector distance of 175 mm at an oscillation angle of 1° per image using a MAR345 detector. The data were processed and scaled using *MOSFLM* and *SCALA* from the *CCP*4 suite, respectively (Winn *et al.*, 2011 ▶). The final statistics of data collection and processing are given in Table 1 ▶.

#### 2.2.3. Structure solution and refinement   

Molecular replacement (MR) was attempted by searching for a single molecule in the asymmetric unit using *Phaser* (McCoy *et al.*, 2007 ▶). Monomers of mAC (PDB entry 1cjk; Tesmer *et al.*, 1999 ▶) and the triple mutant KFD→ERC of Rv1625c (PDB entry 1yk9; Ketkar *et al.*, 2006 ▶) that crystallized as a monomer were used as search models, but no solution was obtained. During this time, the crystal structure of a GC from *C. reinhardtii* (PDB entry 3et6; Winger *et al.*, 2008 ▶) became available. The sequence of GC is 45% identical to the sequence of Rv1625c-F363R, whereas that of mAC is only 35% identical. Hence, MR trials were also carried out with the monomer of GC from *C. reinhardtii*. A unique solution was obtained (*Z*-score = 8.0 and LLG = 66) with this model.

The MR solution was subjected to 20 cycles of rigid-body refinement followed by ten cycles of restrained refinement using *REFMAC*5 from the *CCP*4 suite (Murshudov *et al.*, 2011 ▶). Manual model building was carried out using *Coot* (Emsley *et al.*, 2010 ▶) followed by restrained refinement using *REFMAC*5, and the model was subsequently subjected to automated model building using *ARP*/*wARP* (Perrakis *et al.*, 1999 ▶) followed by many cycles of manual building and restrained refinement. Ethylene glycols were added manually based on a careful examination of the electron-density map. Addition of water molecules and further refinement yielded an *R* factor and *R*_free _of 17.9 and 23.3%, respectively. The final refinement statistics are given in Table 1 ▶.

### 2.3. Rv1625c-Wt   

#### 2.3.1. Crystallization   

**Figure 1:**
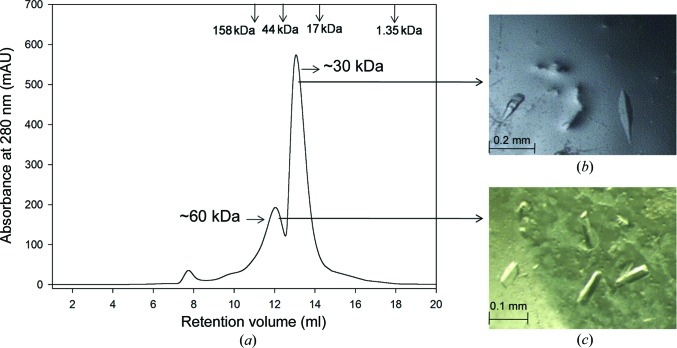
(*a*) Gel-filtration analysis of Rv1625c-Wt shows that it exists predominantly as a monomer (∼30 kDa) with a small fraction of dimer (∼60 kDa). (*b*, *c*) Crystals of Rv1625c-Wt obtained during initial screening of (*b*) the monomeric fraction and (*c*) the dimeric fraction.

Rv1625c-Wt eluted predominantly as a monomeric species on gel filtration (Fig. 1 ▶ *a*) and this monomeric fraction was initially used for crystallization. Crystallization trials were performed with the His-tagged protein. No crystals were obtained, probably owing to the flexible nature of the long N-terminal region. Hence, 23 residues at the N-terminus, including the His tag, were removed from the protein using TEV protease. Rv1625c-Wt devoid of His tag was used in further crystallization trials. To stabilize the dimeric form of Rv1625c-Wt, the metal ion Mg^2+^ (10 m*M* MgCl_2_) and the substrate analogue cordycepin (3′-deoxy­adenosine) were added. The metal ion was added during purification, while the inhibitor was added in a ten-molar excess to the protein prior to crystallization. Small crystals were obtained after mixing 2 µl protein solution with 2 µl crystallization condition consisting of 0.2 *M* ammonium sulfate, 0.1 *M* Tris pH 8.5, 25% PEG 3350 using the microbatch method. This condition was optimized in order to obtain diffraction-quality crystals (Fig. 1 ▶ *b*). The lower amount of dimer obtained from gel filtration (Fig. 1 ▶ *a*) was subsequently concentrated and used for crystallization without TEV protease treatment.

#### 2.3.2. Data collection   

One of the crystals of the monomeric fraction obtained in the optimized condition diffracted to a resolution of 3 Å at the home source. 120 frames were collected with a crystal-to-detector distance of 275 mm using a MAR345 detector. The same crystal was also subjected to X-ray diffraction at a synchrotron source on ESRF beamline 14, where it diffracted to 2.7 Å resolution. 360 frames with an oscillation angle of 1° per image were collected with a crystal-to-detector distance of 270 mm using a MAR Mosaic 225 CCD detector. Data were processed and scaled using *MOSFLM* and *SCALA* from the *CCP*4 suite, respectively (Winn *et al.*, 2011 ▶). The data-collection and processing statistics are given in Table 2 ▶.

#### 2.3.3. Structure solution and refinement   

Molecular replace­ment was carried out using the structure of Rv1625c-F363R as a search model. A clear solution with two molecules in the asymmetric unit was obtained using *Phaser* (*Z* = 27.4 and LLG = 639). During the initial stages of model building, a continuous density between the two subunits was observed. Even after several rounds of model building and refinement, the continuous density persisted. It was realised that this density appeared because of extensive domain swapping between the subunits and that the two chains were connected across the subunits. Since the data showed the presence of twinning (twin operator *k*, *h*, −*l* and twin fraction = 0.42), twin correction, as available in *REFMAC*5.5, was applied in all refinement cycles. The final *R* factor and *R*_free_ were 20.0 and 25.4%, respectively (Table 2 ▶).

Crystals of the dimeric fraction were obtained using the initial screening condition consisting of 0.2 *M* lithium sulfate, 0.1 *M* Tris pH 8.5, 25% PEG 4000 (Fig. 1 ▶ *c*). One of the crystals diffracted to 2.4 Å resolution at the synchrotron source. The data-collection and processing statistics are given in Table 2 ▶. The structure was solved following the procedure that was used to obtain the structure of the monomeric fraction. The final *R* factor and *R*_free_ are 19.6 and 25.9%, respectively (Table 2 ▶). Twinning was not detected in this data set.

## 3. Results and discussion   

### 3.1. Rv1625c-F363R   

The single mutant eluted predominantly as a dimer in gel filtration (Supplementary Fig. S1*a*) which showed much lower activity (63 ± 13 pmol cAMP min^−1^ mg^−1^) compared with the wild-type enzyme (199 ± 30 nmol cAMP min^−1^ mg^−1^) (Ketkar *et al.*, 2006 ▶; Shenoy *et al.*, 2003 ▶). The structure solved by MR has one monomer in the asymmetric unit. It cannot form crystallographic dimers as the protein crystallized in space group *P*2_1_2_1_2_1_, thus indicating that the protein crystallized as a monomer in spite of being a dimer in solution. The only other AC that crystallized as a monomer is the trypano­somal enzyme (Bieger & Essen, 2001 ▶). It was observed that there was a difference in the pH of the buffer used for gel filtration (pH 7.4), at which the enzyme is active, and the pH at which it crystallized (pH 5.5). Thus, it appears that Rv1625c-F363R exists as a monomer at a pH lower than the active pH. However, our attempts to confirm the presence of monomers in solution by gel filtration were not successful since the protein was found to precipitate at the acidic pH of 5.5, close to its pI of 5.7.

#### 3.1.1. Crystal structure of Rv1625c-F363R   

**Figure 2:**
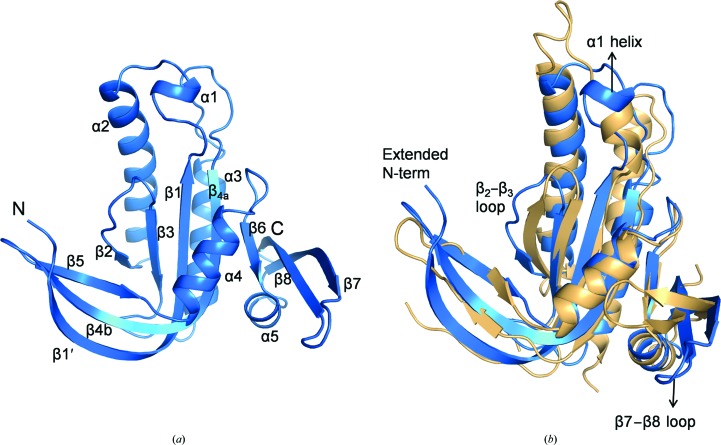
Comparison of the Rv1625c-F363R structure with that of mAC (PDB entry 1cjk). (*a*) Cartoon representation of the Rv1625c-F363R structure (blue) with the secondary-structural elements labelled. (*b*) The Rv1625c-F363R structure (blue) superposed on that of mAC (light orange). The regions of Rv1625c-F363R present in different conformations compared with those of mAC are labelled. Images of the structures shown in Figs. 2 to 7 and Supplementary Figs. 2 and 3 were prepared with *PyMOL* (DeLano, 2002 ▶).

Clear electron density was observed for the region Glu237–Lys425, with 42 and 18 residues missing at the N- and C-termini, respectively. The overall fold of the monomer is similar to other reported class III ACs with the conserved β1–α1–α2–β2–β3–α3–β4 core. The N-terminal residues form an extended β-strand by hydrogen bonding to the β-strands of the dimerization arm to give a three-stranded β-sheet (β1′–β4b–β5; Fig. 2 ▶ *a*). The β7 and β8 strands along with the interconnecting loop have moved dramatically away from the core of the molecule. This region is also referred to as an ‘arm’ subdomain (Kamenetsky *et al.*, 2006 ▶). The α1 helix was found to be shorter than that in mACs (Fig. 2 ▶ *b*).

**Figure 3:**
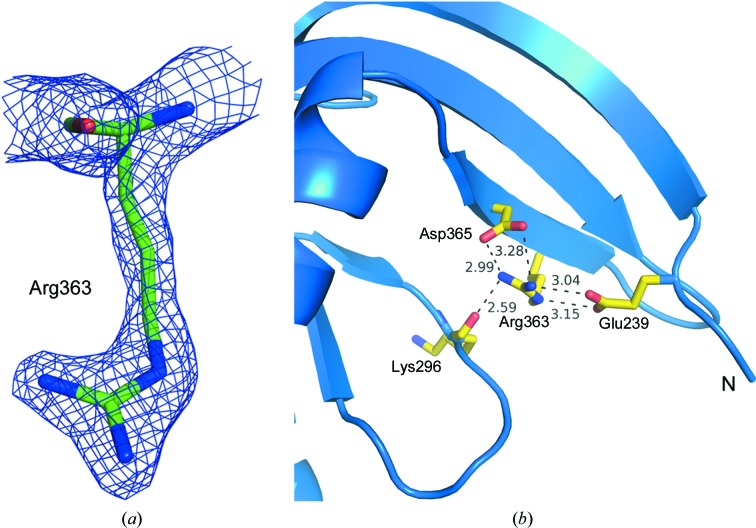
The mutated residue Phe363Arg in Rv1625c-F363R. (*a*) Arg363 is shown in stick representation with the 2*F*_o_ − *F*_c_ electron-density map contoured at the 1.5σ level. (*b*) Residues involved in salt-bridge and hydrogen-bond interactions with Arg363 are shown in stick representation.

**Figure 4:**
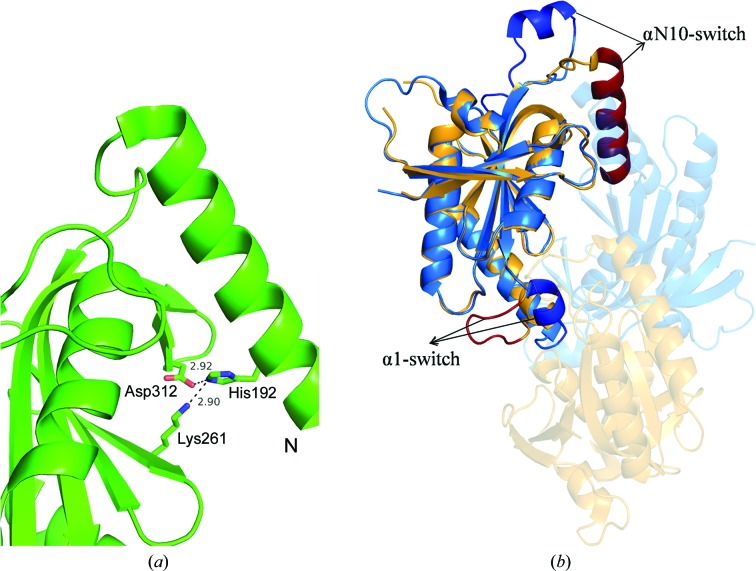
(*a*) Hydrogen-bond interaction of the N-terminal histidine residue of Rv1264 with the substrate-specifying residues. (*b*) Active (blue) and inactive (yellow–orange) forms of Rv1264. The subunit which moves from the inactive state to the active state is shown with 60% transparency. The arrows show the switch elements α1-switch and αN10-switch of the active and inactive forms, which are highlighted in darker shades.

The density for the mutated residue Arg363 could be seen clearly (Fig. 3 ▶ *a*). Arg363 forms salt bridges with the N-terminal residue Glu239 and the substrate-specifying residue Asp365 (Fig. 3 ▶ *b*). Because of this salt-bridge interaction between the N-terminal Glu239 and the mutated Arg363 residue, the N-terminal segment is pulled towards the dimer-interface region. When the monomers of Rv1625c-F363R are individually superposed on different subunits of dimeric ACs or GCs, the extended N-terminal segment of one monomer was found to make steric clashes with the residues of helix α2 and strand β2 of the other subunit. Thus, the extended region of the N-terminus of this structure, stabilized by the mutated residue, hinders dimer formation. Interestingly, these interactions appear to resemble those occurring at a spatially equivalent location in the inactive form of Rv1264, another AC present in *M. tuberculosis*. As shown in Fig. 4 ▶(*a*), a histidine residue in the N-terminal α-helix, called the αN10-switch, of the regulatory domain of Rv1264 is found in a position similar to Glu239 of the N-terminal segment of Rv1625c (Tews *et al.*, 2005 ▶). This histidine residue interacts with the catalytic aspartate. Owing to a conformational change in this αN10-switch region, it occupies a different position from that in the active state of the enzyme, preventing the formation of the head-to-tail functional dimer in the inactive form (Fig. 4 ▶ *b*).

The conformation of the β2–β3 loop of Rv1625c-F363R was found to be different when compared with that in mAC (Fig. 2 ▶ *b*). The β2–β3 loops meet at the centre of the dimeric interface region and contribute to dimer stabilization (Sinha & Sprang, 2006 ▶). Because of this altered conformation, the side chain of Ser298 present in this region makes steric clashes with the β2–β3 loop of the other protomer in the modelled dimer of Rv1625c-F363R.

The β7–β8 loop was found to interact with a symmetry-related molecule. The β3′–α3′ loop (where ′ indicates residues of a symmetry-related molecule) was found to be placed between the β7–β8 loop and the α4 helix (Supplementary Fig. S2*a*) and thus prevents the arm subdomain from adopting the conformation which is seen in other ACs. This β7–β8 loop conformation is stabilized by numerous van der Waals interactions with the symmetry-related molecule and is further stabilized by the hydrogen bond between His407 and Asp399′ of the β3′–α3′ loop (Supplementary Fig. S2*b*). The α1 helix was found to interact with the α2′ helix by forming (i) salt bridges from Arg263 to Asp288′ and Glu285′ and (ii) a hydrogen bond between Thr261 and Glu285′. These interactions (Supplementary Fig. S2*b*) make the α1-helix shift from its position compared with mAC (Fig. 2 ▶ *b*).

#### 3.1.2. The active site   

The side-chain conformations of some of the substrate-binding residues in this monomeric structure appear to be different from those observed in the active forms of the other reported structures. These side chains are likely to acquire conformations that are optimal for substrate binding in the active form of the enzyme, as observed in the case of human sAC (Kleinboelting *et al.*, 2014 ▶) and mAC (Tesmer *et al.*, 1999 ▶).

**Figure 5:**
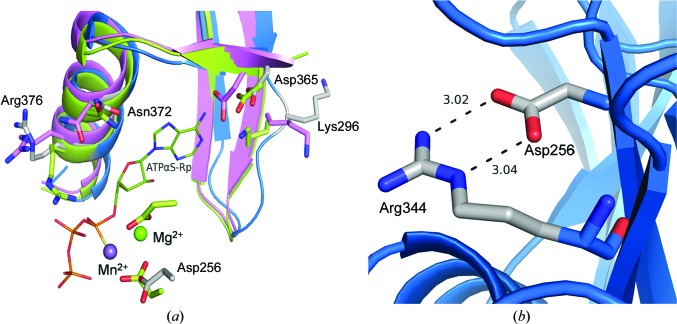
(*a*) Comparison of the active-site residues of Rv1625c-F363R (blue) with active (PDB entry 1cjk; yellow) and inactive (PDB entry 1ab8; pink) forms of mAC. The active-site residues of Rv1625c-F363R are shown in grey and labelled. The substrate analogue ATPαS-Rp and metal ions bound in the active mAC structure are shown in stick and sphere representations, respectively. (*b*) Salt bridges formed by the catalytic aspartate Asp296 with Arg344 are shown.

The side chain of the substrate-specifying residue Lys296 is in a different orientation (Fig. 5 ▶ *a*) when compared with its mammalian counterpart that was bound to the substrate analogue (active form). The interaction of the carbonyl group of Lys296 with the side chain of the mutated residue Arg363 might be causing this altered orientation (Fig. 3 ▶ *b*). The other substrate-binding residue Asp365 is in a similar conformation to its counterpart in active mammalian ACs.

Electron density for the side chain of one of the metal-binding residues, Asp300, was not observed. The second metal-binding residue, Asp256, has a minor conformational change compared with that of the active mammalian enzyme (Figs. 5 ▶ *a* and 5 ▶ *b*). This conformation of the aspartate is stabilized by a salt bridge to the side chain of Arg344.

The residue Arg376, the predicted function of which is to bind the negatively charged phosphate group of ATP, has its side chain in a different orientation (Fig. 5 ▶ *a*) that is not optimal for binding to the phosphate group of the substrate. A similar orientation of this transition-state residue arginine is seen in the case of the inactive mAC C2–C2 homodimer. The ribose-orienting transition-state-stabilizing residue Asn372 is in a similar orientation (Fig. 5 ▶ *a*) to Asn1029 of the active mACs (PDB entry 1cjk).

### 3.2. Rv1625c-Wt: the domain-swapped dimer   

#### 3.2.1. Form 1   

Rv1625c-Wt eluted predominantly as a monomer in gel filtration (Fig. 1 ▶ *a*) and this monomeric fraction was initially used for crystallization. Two subunits (*A* and *B*) were obtained in the asymmetric unit with extensive domain swapping. Clear electron density is observed for residues 240–426 of chain *A* (except for the β1–α2, α3–β4 and β7–β8 loops) and for residues 240–428 of chain *B* (except for the β1–α2 and β7–β8 loops). All of the residues for which electron density was not observed belong to loop regions of the molecule.

**Figure 6:**
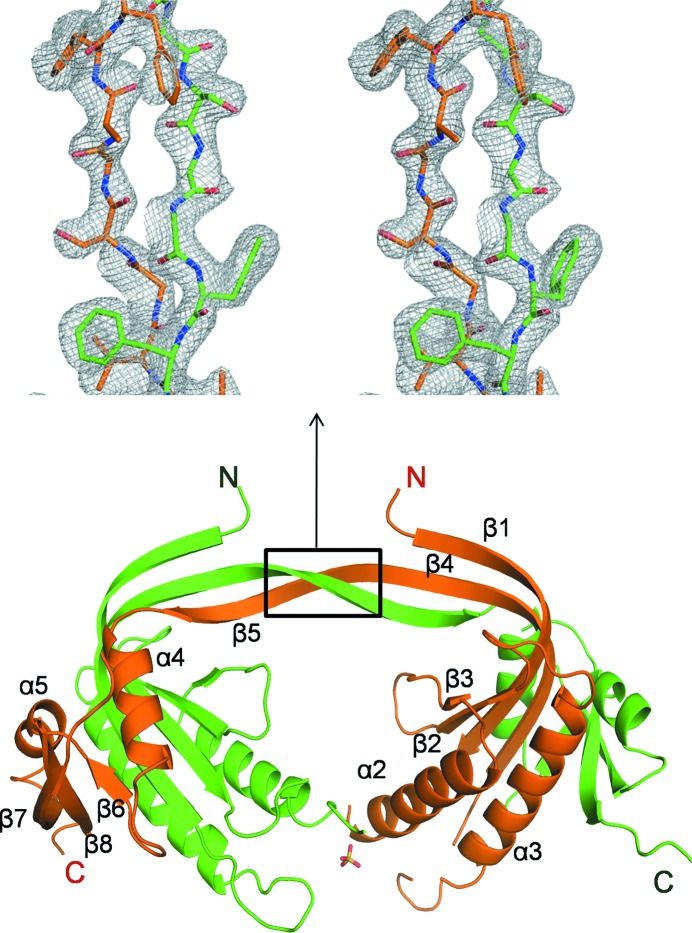
Domain-swapped dimer of Rv1625c-Wt. The two subunits are coloured differently. Secondary-structural elements are labelled for one of the subunits of Rv1625c-Wt. An OMIT map contoured at the 2.5σ level is shown where the domain swapping occurred. (This view, which is different from that shown in the box, was chosen for clarity.)

Rv1625c-Wt crystallized as a domain-swapped dimer. In general small regions are involved in domain swapping, but in this case about half of the residues (β5–α4–β6–α5–β7–β8) are swapped between the subunits, leading to a large number of interactions between the subunits (Fig. 6 ▶). The dimer has a head-to-head arrangement instead of the head-to-tail arrangement which is seen in the case of dimeric active ACs or GCs. The intact substrate-binding pockets at the active dimer interface are not formed in the swapped dimer. Metal ions and the substrate analogue cordycepin added during crystallization were not seen in the structure because of the altered domain arrangement that is not conducive for binding ligands. Thus, this form of Rv1625c-Wt is an inactive form in spite of being a dimer. Density for the α1 helix was also not seen, indicating that it was disordered in the structure.

Domain swapping is a frequent phenomenon in oligomeric proteins where one or more structural elements are exchanged between two identical subunits, keeping the overall globular form of the domains unaltered. Several examples of domain swapping can be found in protein crystal structures (Carey *et al.*, 2007 ▶; Gronenborn, 2009 ▶; Shingate & Sowdhamini, 2012 ▶). In a number of cases proline residues were found to occur in hinge loops where swapping often takes place. A few residues at such hinge regions appear to have considerable strain which is released upon domain swapping, rendering more favourable conformations of these residues. In the case of Rv1625c-Wt the dimerization-arm loop (β4–β5 loop) opened up and exchanges the part of the molecule consisting of the β5–α4–β6–α5–β7–β8 structural elements with the other subunit (Fig. 6 ▶). Consequently, a new open interface is formed between the α2 helices of each subunit.

A sulfate ion found in the new open interface neutralizes the positive charge present at the interface, interacting with the arginine side chains from both subunits (Supplementary Fig. S3). The open interface is also stabilized by a hydrogen bond formed between Arg274 of chain *A* and Ser281 of chain *B*.

**Figure 7:**
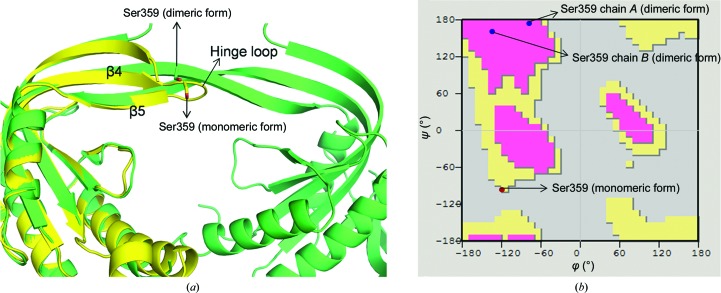
(*a*) The Rv1625c-F363R structure (yellow) superposed on the domain-swapped dimer of Rv1625c-Wt (green). Ser359 is shown in stick representation in both of the structures. (*b*) Ser359 in the structures of Rv1625c-F363R (red dot) and Rv1625c-Wt (blue dots) is depicted in the Ramachandran plot.

There is no proline residue in the hinge-loop region (β4–β5 loop) where the domain swapping takes place. The residue Ser359 present in the hinge loop of Rv1625c-F363R (Fig. 7 ▶ *a*), with ϕ = −123.4°, ψ = −93.0°, falls at the edge of the allowed region of the Ramachandran plot (Fig. 7 ▶ *b*). In the Rv1625c-Wt structure, Ser359 in chain *A* has ϕ = −76.9°, ψ = 175.2° and that in chain *B* has ϕ = −144.6°, ψ = 168.9°, which fall in the favourable regions of the Ramachandran plot (Fig. 7 ▶ *b*). Thus, this serine residue with a slightly unfavourable conformation possibly creates a strain in the hinge region which is released upon formation of the swapped dimer.

Domain swapping can be triggered by changes in external conditions such as solubility and pH. When the solubility of the protein decreases, the protein might open up to release the strain present in the hinge region, allowing the polypeptide chain beyond the hinge to detach from the subunit and interact with another subunit, forming new interactions. This is a slow process since an energy barrier has to be overcome by breaking interactions within the same subunit (Nagradova, 2002 ▶). This could be the reason for the long time (two months) taken for the formation of crystals of domain-swapped Rv1625c-Wt. It is likely that this strain, along with the crystallization conditions, which cause a decrease in the solubility of the protein, would have caused the domain swapping in the case of Rv1625c-Wt.

Most of the active residues of the Rv1625c-Wt are in a similar conformation to the Rv1625c-F363R active-site residues except for Arg376, which has a conformation close to its counterpart in active AC structures.

Structure-based sequence alignment of Rv1625c-Wt with other nucleotide cyclases shows the conservation of dimer-interface residues, with the exception of Ser298 (Supplementary Fig. S4). This residue is present in the β2–β3 loop that forms the inner edge of the dimer interface. The structurally equivalent residue to Ser298 of Rv1625c in other class III AC is a hydrophobic residue that forms a hydrophobic inter­action with the corresponding residue in the β2–β3 loop of the dimeric partner. This residue thus strengthens the dimer interaction. The residue in this position also contributes to the hydrophobic interaction of the enzyme with the nitrogen base of the substrate. Ser298 of Rv1625c cannot make such interactions and is possibly involved in a different kind of interaction.

#### 3.2.2. Form 2   

Crystallization of the dimeric fraction of Rv1625c-Wt obtained during gel filtration was carried out to check whether it forms a domain-swapped dimer like that described above or a different type of dimer. Surprisingly, this dimeric fraction also crystallized as a domain-swapped dimer that was similar to the swapped dimer formed by the monomeric fraction. Clear electron density is observed for residues 240–428 of chain *A* (except for the β1–α2, β2–β3, α3–β4 and β7–β8 loops) and for residues 240–425 of chain *B* (except for the β1–α2, α3–β4 and α4–β6 loops). Comparison of forms 1 and 2 of Rv1625c-Wt shows that they form similar structures with small differences. When the protomers of each form are superimposed, it was observed that the other protomers are related by a rotation of 19°. Because of this rotation, the α2 helices of the domains come closer in form 2, precluding the space to accommodate a sulfate ion present in form 1. The possible explanation for the observed inactivity of the dimeric fraction is that it exists as a domain-swapped dimer in solution and cannot convert to the active dimeric state owing to strong interactions in the swapped form. The monomeric form, on the other hand, has no such constraints and forms the active dimer upon substrate binding.

In an earlier study, we reported that the dimeric fraction of Rv1625c-Wt did not show any AC activity (Ketkar *et al.*, 2006 ▶). In the absence of the availability of structural information, an attempt was made to explain this lack of activity by speculating that the protomers of the dimer might not be in the correct juxtaposition required for catalysis as in the case of the inhibited form of Rv1264 (Tews *et al.*, 2005 ▶). The current study showing the presence of the swapped dimer with an entirely new mode of dimerization which completely breaks the active-site architecture not only provides an explanation for our earlier results but is also consistent with our hypothesis about the lack of activity of the dimer.

To check for monomer–dimer transitions and their stabil­ities, we collected the monomeric and dimeric fractions separately and performed gel-filtration experiments on each of them. As expected, the monomeric state showed a transition to the dimeric state (Supplementary Fig. S5), whereas the dimeric form was found to be stable over time. The appearance of both fractions initially indicates that dimers are also produced during expression of the recombinant protein. In the presence of substrates and the required metals, the monomers convert to active dimers transiently. The dimers, whether coexisting with the monomers or formed from the monomers, have the same structure as the swapped dimers with no activity.

**Figure 8:**
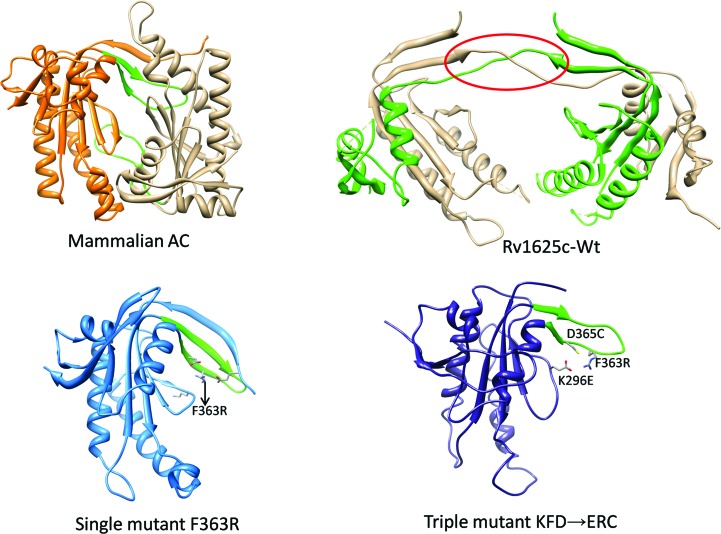
Comparison of the structures of Rv1625c-Wt and its mutants Rv1625c-F363R and KFD→ERC with mAC. The dimerization-arm region is shown in green in each of the structures except for Rv1625c-Wt, in which it is highlighted with a circle. This figure was prepared with UCSF Chimera (Petterson *et al.*, 2004 ▶).

The two structures reported here along with the triple-mutant structure KFD→ERC reported previously (Ketkar *et al.*, 2006 ▶) have different conformations of the dimerization-arm region that are also different from the conformation of this region in active mAC (Fig. 8 ▶). In the case of the mutant proteins, the mutated residues present in the dimerization-arm region stabilize the altered conformations of this loop and the β2–β3 loop (Fig. 2 ▶ *b*), both of which are involved in dimer stabilization (Sinha & Sprang, 2006 ▶). Similar to the triple mutant, the residues present in the β2–β3 loop of Rv1625c-F363R also make steric clashes with the subunit when superposed on the dimeric AC structure in addition to the severe steric clashes made by the extended N-terminal loop. The hinge loop of Rv1625c-Wt involved in domain swapping is also present in the dimerization-arm region. These observations imply that the N-terminal segment, the dimerization arm and the β2–β3 loop play crucial roles in the oligomerization of the enzyme, thereby affecting its activity.

Some of the reported crystal structures of cyclases suggest that the head-to-tail dimers of the enzymes adopt different strategies to shift from the inactive to the active form. These strategies include side-chain conformational changes in the binding-site residues (Kleinboelting *et al.*, 2014 ▶; Tesmer *et al.*, 1997 ▶, 1999 ▶), shifts in α-helices and loops (Steegborn *et al.*, 2005 ▶; Winger *et al.*, 2008 ▶) and rotation of the entire subunit (Sinha *et al.*, 2005 ▶; Tesmer *et al.*, 1997 ▶; Allerston *et al.*, 2013 ▶). The two crystal structures reported here indicate that the mechanism of conversion between the active and inactive forms is different in the case of Rv1625c.

## 4. Conclusions   

Crystal structures of Rv1625c and its single mutant Rv1625c-F363R have been determined. Both of them crystallized in inactive forms. The mutant crystallized as a monomer even though it exists as a dimer in solution. The extended N-terminus of the mutant, stabilized by the mutated residue Phe363Arg, hinders dimer formation. The wild-type enzyme exists predominantly as a monomer in solution but crystallized as a domain-swapped dimer. The geometry of the active site is lost in the head-to-head arrangement of the domain-swapped dimer, rendering it inactive. The minor inactive dimeric fraction in solution also has a similar swapped arrangement in the crystalline state. These observations lead to the conclusion that the transition of the monomeric enzyme to the active dimeric form takes place upon activation, whereas the preformed dimers in solution cannot be triggered to become functional dimers by the mere addition of the substrate. At this stage the biological significance of the swapped dimers is unknown. It is clear that the swapped dimers cannot perform the adenylyl cyclase activity but it can be speculated that they might play a different role such as interaction with other molecules. The crystal structures presented here also provide new insights into the inherent flexibility of the molecule, which is likely to be related to its function. The function of the molecule appears to be regulated by the N-terminal region, the dimerization arm and the β2–β3 loop, which affect the oligomeric nature of the enzyme.

Guo *et al.* (2001 ▶) observed that at higher concentrations of the catalytic domain of Rv1625c, the presence of dimers and other higher oligomeric states increases, causing reduced activity. It is not possible to guess whether these dimers were formed by the swapped catalytic domains, but one cannot rule out the possibility that these could be swapped dimers in the light of the present study. In the full-length protein with an additional six transmembrane helices, the linker region between the last helix and the catalytic domain is about 40 residues in length and can easily facilitate formation of either the head-to-tail domain or the swapped domain. However, determination of the nature of the dimers *in vivo*, whether swapped or in the active head-to-tail form, may not be trivial but can be attempted with techniques such as single-molecule fluorescence resonance energy transfer (smFRET). This method has successfully been used to distinguish between two conformational states of a recombinant SNARE protein (Sakon & Weninger, 2010 ▶). As it has been shown that full-length Rv1625c could be targeted to the cell membrane of HEK293 cells (Guo *et al.*, 2001 ▶), it is possible to specifically label the protein by cysteine mutations at appropriate positions with acceptor and donor molecules using the available information from the crystal structures of both active and swapped forms of the dimers. These labelled molecules can then be microinjected into the cell and the resulting amount of FRET signal could be examined to distinguish between the type of oligomers formed in the cell.

The coordinates and structure factors have been deposited in the Protein Data Bank with accession codes 4p2m and 4p2x for Rv1625c-Wt forms 1 and 2, respectively, and 4p2f for Rv1625c-F363R.

## Supplementary Material

PDB reference: Rv1625c, wild type, 4p2m

PDB reference: 4p2x

PDB reference: F363R mutant, 4p2f

Supplementary files. DOI: 10.1107/S2052252514016741/jt5004sup2.pdf

## Figures and Tables

**Table 1:** Data-collection and refinement statistics for Rv1625c-F363R Values in parentheses are for the outer shell.

Data collection	
Wavelength (Å)	1.54
Space group	*P*2_1_2_1_2_1_
*a*, *b*, *c* (Å)	29.89, 67.37, 101.55
α, β, γ (°)	90, 90, 90
Resolution range (Å)	40.48–2.05 (2.10–2.05)
No. of unique reflections	13415 (1896)
Completeness (%)	98.98 (97.65)
Multiplicity	6.6 (6.3)
〈*I*/σ(*I*)〉	19.8 (4.3)
*R*_r.i.m._ †	0.083 (0.493)
Refinement	
Resolution range (Å)	40.48–2.05 (2.10–2.05)
No. of reflections	
Working set	12712 (914)
Test set	663 (43)
Final *R*_cryst_	0.179 (0.218)
Final *R*_free_	0.233 (0.274)
No. of non-H atoms	
Protein	1454
Ligand	85
Solvent	92
Total	1631
R.m.s. deviations	
Bonds (Å)	0.013
Angles (°)	1.728
Average *B* factors (Å^2^)	
Protein	24.6
Ligand	40.9
Water	34.8
Ramachandran plot	
Most favoured (%)	96.8
Allowed (%)	3.2

† Estimated *R*_r.i.m._ = *R*_merge_[*N*/(*N* − 1)]^1/2^, where *N* is the data multiplicity.

**Table 2:** Data-collection and refinement statistics for Rv1625c-Wt Values in parentheses are for the outer shell.

	Form 1	Form 2
Data collection		
Wavelength (Å)	0.976	0.978
Space group	*P*6_5_	*P*6_5_
*a*, *b*, *c* (Å)	74.57, 74.57, 133.74	75.57, 75.57, 133.17
α, β, γ (°)	90, 90, 120	90, 90, 120
Resolution range (Å)	46.45–2.70 (2.85–2.70)	37.78–2.4 (2.53–2.40)
No. of unique reflections	11639 (1690)	16832 (2464)
Completeness (%)	100 (100)	99.8 (99.6)
Multiplicity	23 (23)	11.5 (11.5)
〈*I*/σ(*I*)〉	21.4 (7.1)	23.5 (4.9)
*R*_r.i.m._ †	0.142 (0.568)	0.084 (0.550)
Refinement		
Resolution range (Å)	37.28–2.70 (2.77–2.70)	36.76–2.40 (2.46–2.40)
No. of reflections		
Working set	11085 (838)	15982 (1185)
Test set	513 (31)	849 (61)
Final *R*_cryst_	0.200 (0.267)	0.196 (0.219)
Final *R*_free_	0.254 (0.329)	0.260 (0.297)
No. of non-H atoms		
Protein	2529	2547
Ion	10	7
Ligand	63	28
Solvent	65	53
Total	2667	2635
R.m.s. deviations		
Bonds (Å)	0.006	0.007
Angles (°)	1.178	1.151
Average *B* factors (Å^2^)		
Protein	34.3	40.0
Ion	62.6	38.5
Ligand	58.6	64.1
Water	29.1	37.3
Ramachandran plot		
Most favoured (%)	96.7	98.1
Allowed (%)	2.7	1.9
Outliers (%)	0.6	

† Estimated *R*_r.i.m._ = *R*_merge_[*N*/(*N* − 1)]^1/2^, where *N* is the data multiplicity.
