# Supplementary material for: New structural forms of a mycobacterial adenylyl cyclase Rv1625c
Source: IUCrJ. 2014 Aug 22;1(Pt 5):338–48. doi: 10.1107/S2052252514016741 (PMC4174876; doi:10.1107/S2052252514016741)
Supplement: Supplementary file 1 [file m-01-00338-sup2.pdf]

# IUCrJ

**Volume 1 (2014)**


**Supporting information for article:**

## New structural forms of a mycobacterial adenylyl cyclase Rv1625c

**Deivanayaga Barathy, Rohini Mattoo, Sandhya Visweswariah and Kaza Suguna**

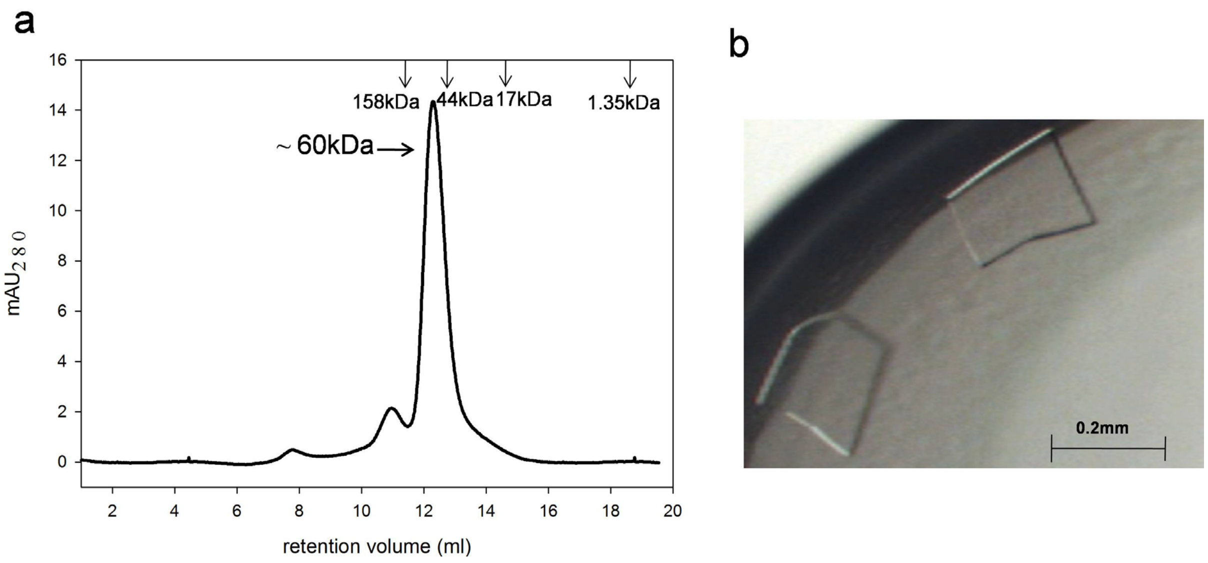


**Figure S1** (a) Gel filtration analysis of Rv1625c-F363R shows the presence of dimer (~60 kDa). (b) Crystals of Rv1625c-F363R obtained after optimization.

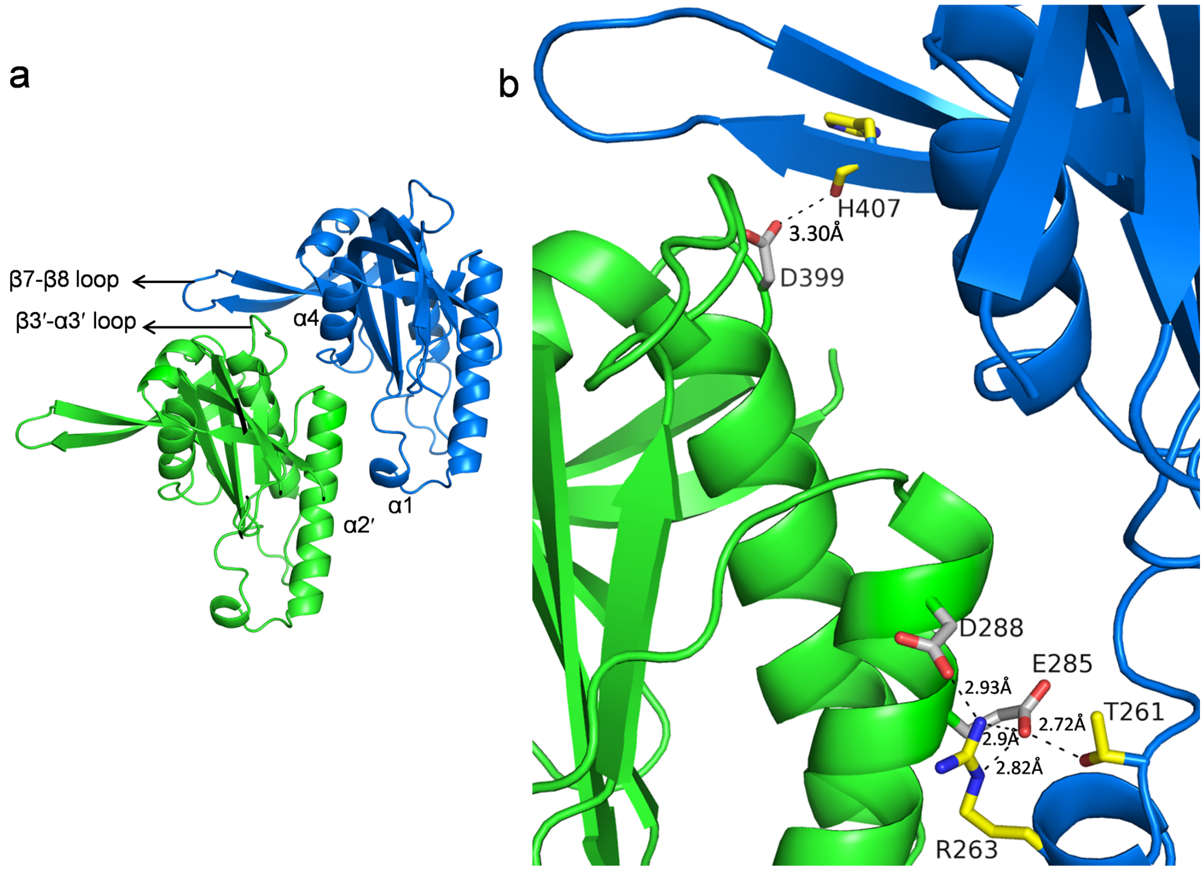


**Figure S2** (a) The proximity of β3′-α3′ loop region of the symmetry-related molecule (green) to the β7-β8 loop and α4 helix of Rv1625c-F363R (blue) is shown. (b) The residues forming salt bridge and H-bonds between Rv1625c-F363R and its symmetry-related molecule are shown in sticks.

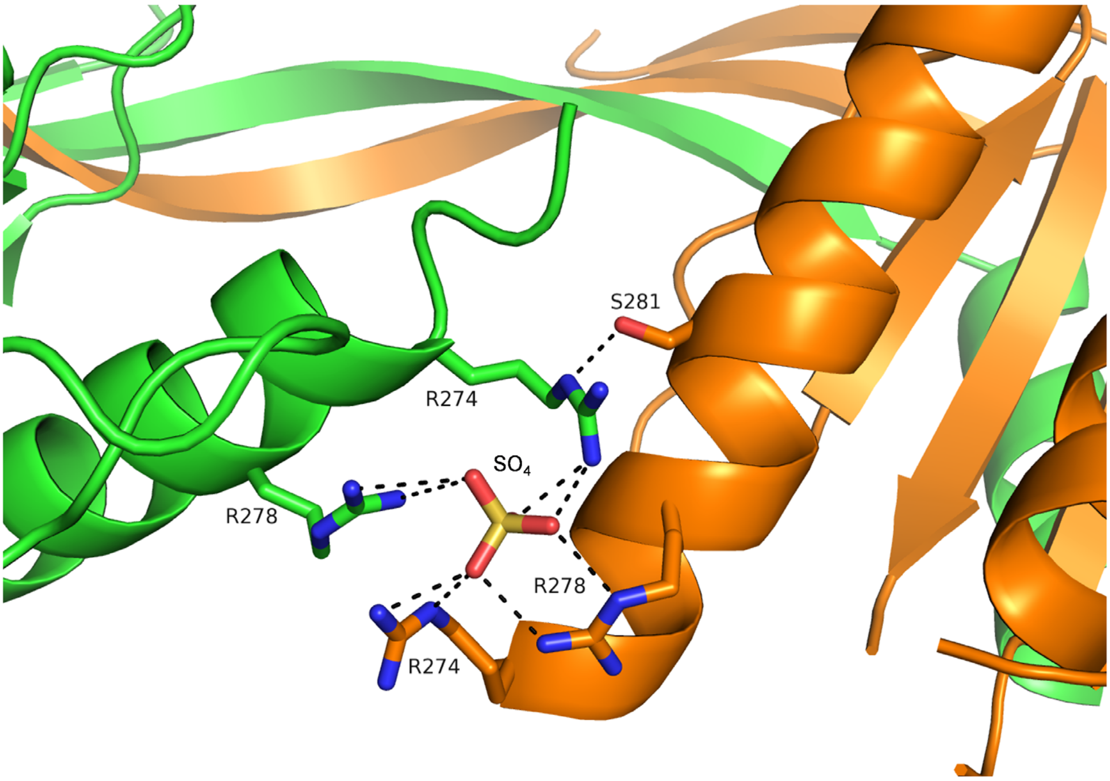


**Figure S3** Interactions at the new interface of Rv1625c-Wt domain swapped dimer**.** Each monomer is coloured differently in orange (chain B) and green (chain A). The interacting residues along with suphate ion are shown in sticks.

```
Rv1625cWt monomer    GAGRLYSAFDALVA---Q--HG-LEKIKVSGDSYMVVSGVPR--PRP--DHTQALADFAL
1cjk_a_chain         TLNELFARFDKLAA---E--NH-CLRIKILGDCYYCVSGLPE--ARA--DHAHCCVEMGM
1cjk_b_chain         LLNEIIADFDDLLS---KPKFSGVEKIKTIGSTYMAATGLSAIR-QY--MHIGTMVEFAY
2W01                 VLNIYFGKMADVIT---H--HG-GTIDEFMGDGILVLFGAPT--SQQ--DDALRAVACGV
3ET6                 LLDELYQRFDAAIE---E--YPQLYKVETIGDAYMVVCNVTV--PCD--DHADVLLEFAL
3R5G                 LLNNYLNEMSKIAL---K--YG-GTIDKFVGDCVMVFFGDPS--TQGAKKDAVAAVSMGI
3UVJa_chain          MLNALYTRFDQQCG---E--LD-VYKVETIGDAYCVAGGLHK--ESD--THAVQIALMAL
3UVJB_chain          LLNDLYTRFDTLTD-SRK--NPFVYKVETVCDKYMTVSGLPE--PCI--HHARSICHLAL
1WC0                 LLNEYLGEMTRAVF---E--NQ-GTVDKFVGDAIMALYGAPE--EMSPSEQVRRAIATAR
1YBT                 LLDNHDTIVCHEIQ---R--FG-GREVNTAGDGFVATFT-----------SPSAAIACAD
1Y10                 LAGRLAGLARDLTAP------P-VWFIKTIGDAVMLVCP-----------DPAPLLDTVL
1FX2                 AVAAHHRMVRSLIG---R--YK-CYEVKTVGDSFMIASK-----------SPFAAVQLAQ
```

**Figure S4** Structure-based sequence alignment of Rv1625c-Wt with different AC and GC catalytic domains**.** The dimer interface of Rv1625c-Wt has a serine substituted in place of the hydrophobic residues found in other cyclases (highlighted in red).

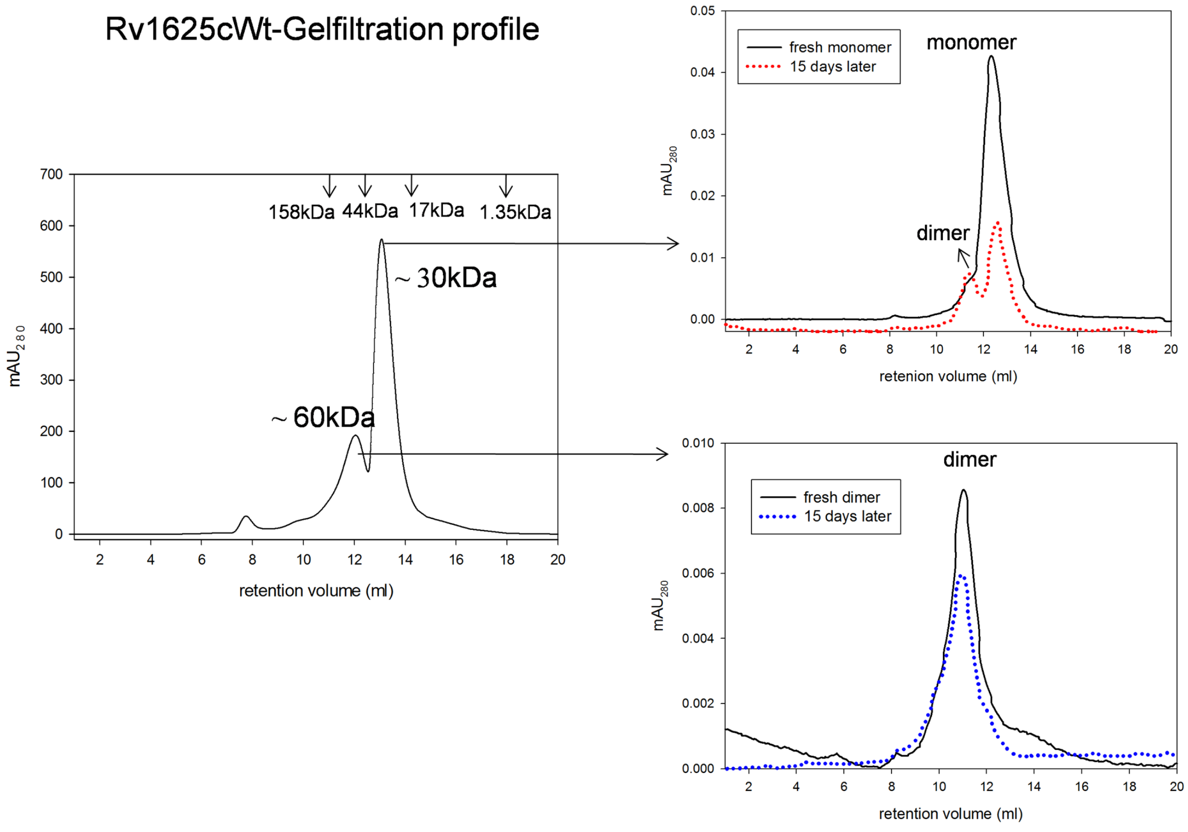


**Figure S5** Gel-filtration studies on monomeric and dimeric fractions of Rv1625c-Wt
